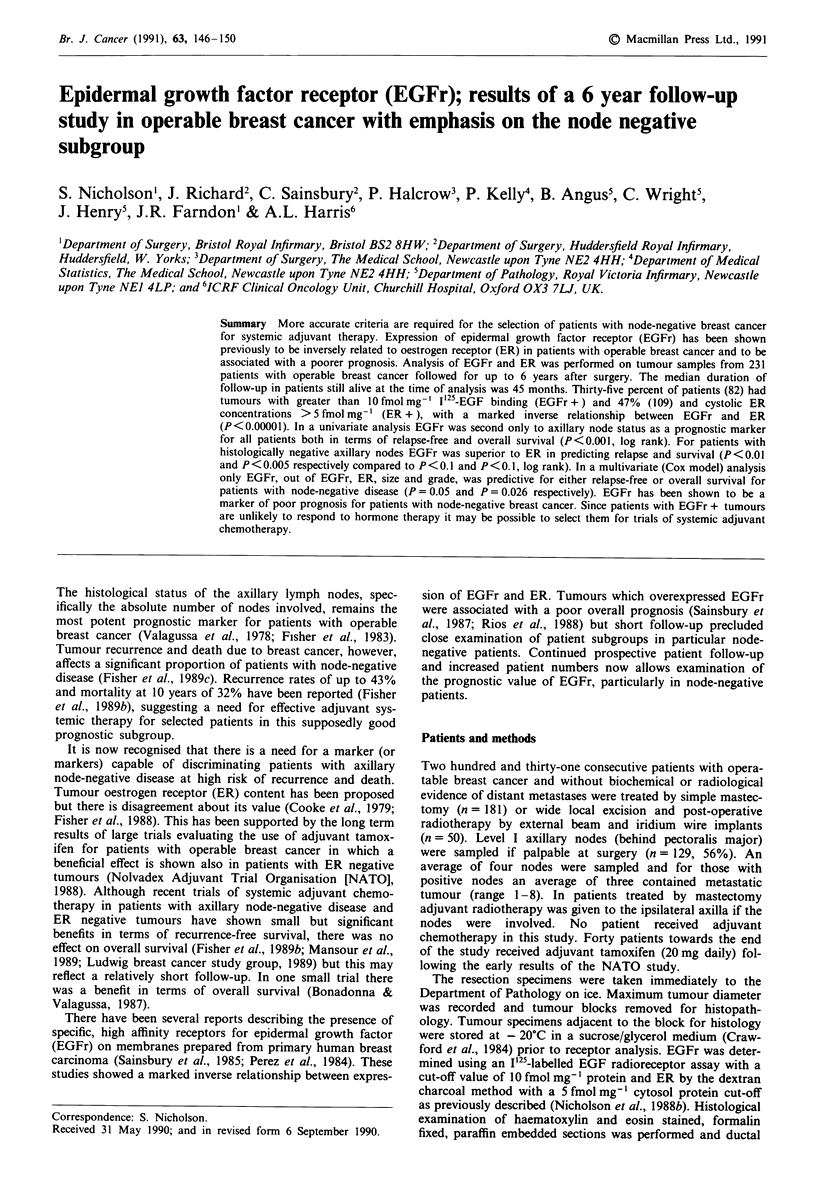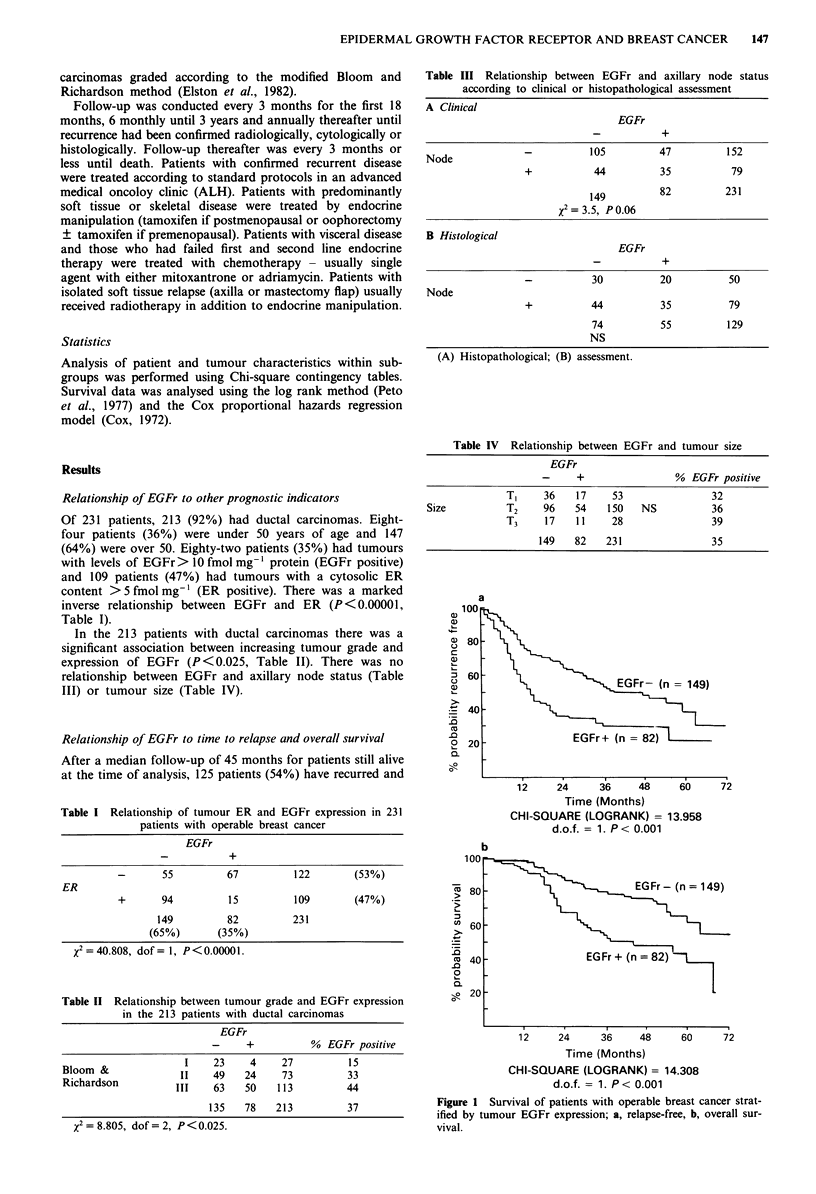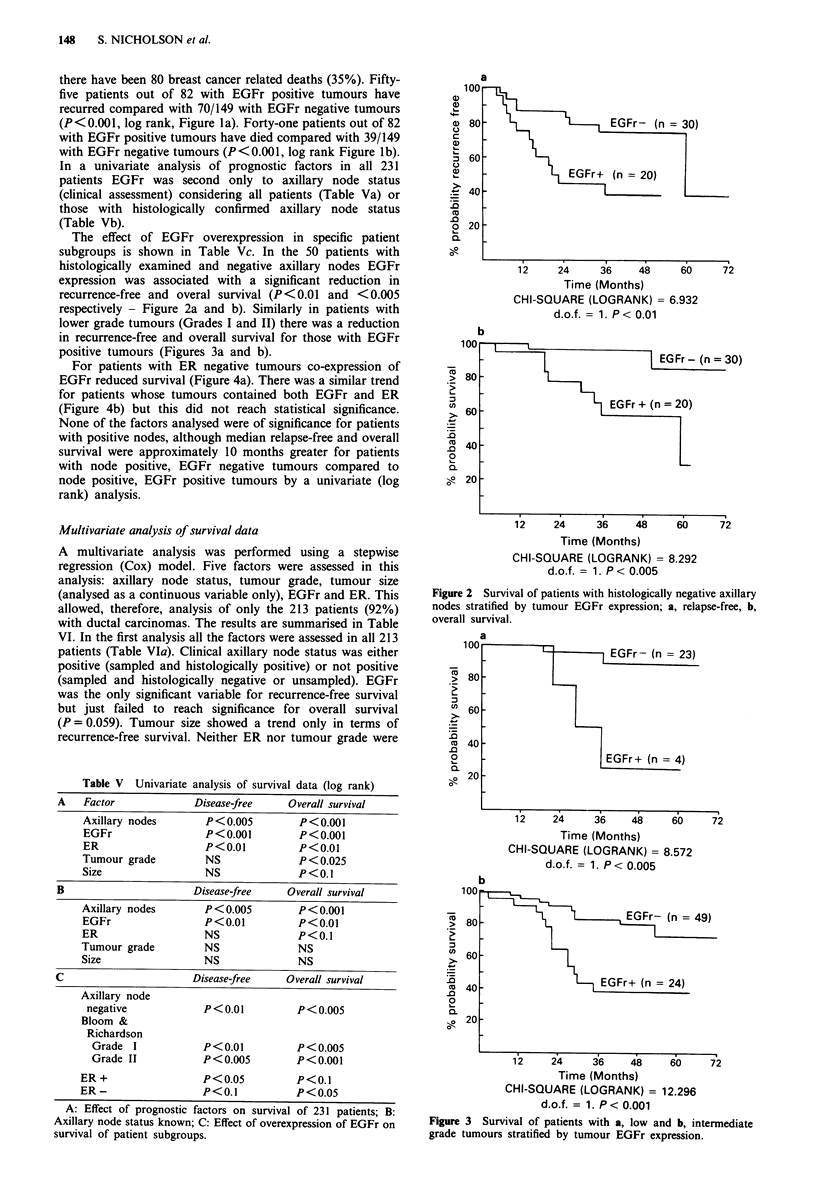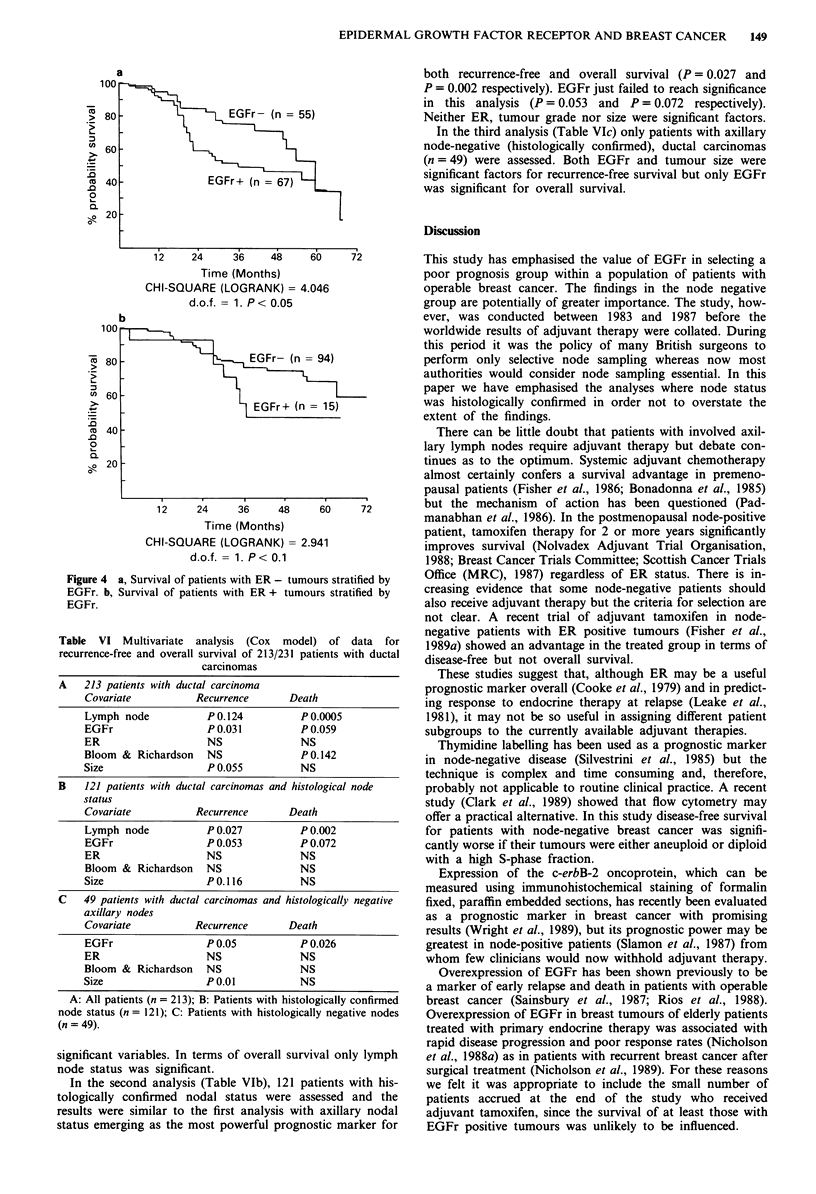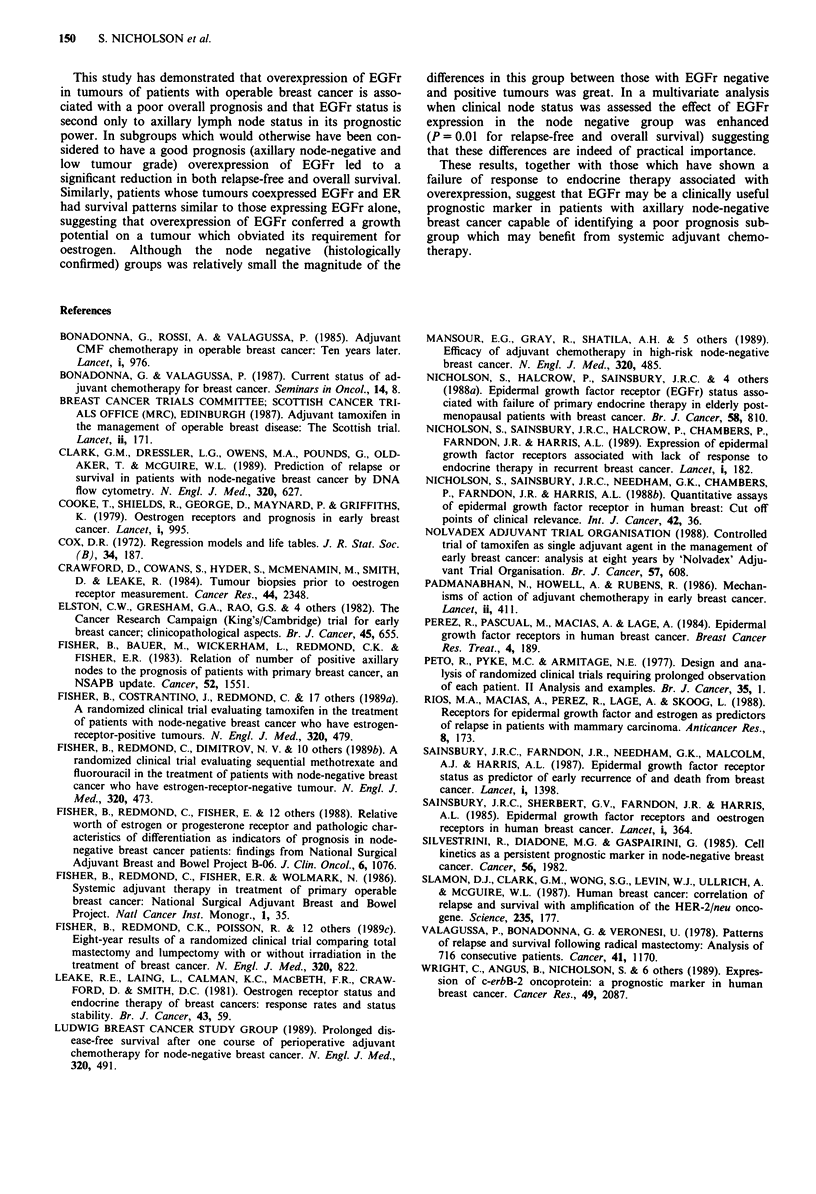# Epidermal growth factor receptor (EGFr); results of a 6 year follow-up study in operable breast cancer with emphasis on the node negative subgroup.

**DOI:** 10.1038/bjc.1991.30

**Published:** 1991-01

**Authors:** S. Nicholson, J. Richard, C. Sainsbury, P. Halcrow, P. Kelly, B. Angus, C. Wright, J. Henry, J. R. Farndon, A. L. Harris

**Affiliations:** Department of Surgery, Bristol Royal Infirmary, W. Yorks, UK.

## Abstract

More accurate criteria are required for the selection of patients with node-negative breast cancer for systemic adjuvant therapy. Expression of epidermal growth factor receptor (EGFr) has been shown previously to be inversely related to oestrogen receptor (ER) in patients with operable breast cancer and to be associated with a poorer prognosis. Analysis of EGFr and ER was performed on tumour samples from 231 patients with operable breast cancer followed for up to 6 years after surgery. The median duration of follow-up in patients still alive at the time of analysis was 45 months. Thirty-five percent of patients (82) had tumours with greater than 10 fmol mg-1 I125-EGF binding (EGFr+) and 47% (109) and cystolic ER concentrations greater than 5 fmol mg-1 (ER+), with a marked inverse relationship between EGFr and ER (P less than 0.00001). In a univariate analysis EGFr was second only to axillary node status as a prognostic marker for all patients both in terms of relapse-free and overall survival (P less than 0.001, log rank). For patients with histologically negative axillary nodes EGFr was superior to ER in predicting relapse and survival (P less than 0.01 and P less than 0.005 respectively compared to P less than 0.1 and P less than 0.1, log rank). In a multivariate (Cox model) analysis only EGFr, out of EGFr, ER, size and grade, was predictive for either relapse-free or overall survival for patients with node-negative disease (P = 0.05 and P = 0.026 respectively). EGFr has been shown to be a marker of poor prognosis for patients with node-negative breast cancer. Since patients with EGFr+ tumours are unlikely to respond to hormone therapy it may be possible to select them for trials of systemic adjuvant chemotherapy.


Br. J. Cancer (1991), 63, 146-150 © Macmillan Press Ltd., 1991

### Epidermal growth factor receptor (EGFr); results of a 6 year follow-up study in operable breast cancer with emphasis on the node negative subgroup

S. Nicholson[1], J. Richard[2], C. Sainsbury[2], P. Halcrow[3], P. Kelly[4], B. Angus[5], C. Wright[5], J. Henry[5], J.R. Farndon[1] & A.L. Harris[6]

[1]*Department of Surgery, Bristol Royal Infirmary, Bristol BS2 8HW;* [2]*Department of Surgery, Huddersfield Royal Infirmary, Huddersfield, W. Yorks;* [3]*Department of Surgery, The Medical School, Newcastle upon Tyne NE2 4HH;* [4]*Department of Medical Statistics, The Medical School, Newcastle upon Tyne NE2 4HH;* [5]*Department of Pathology, Royal Victoria Infirmary, Newcastle upon Tyne NE1 4LP; and* [6]*ICRF Clinical Oncology Unit, Churchill Hospital, Oxford OX3 7LJ, UK.*

**Summary** More accurate criteria are required for the selection of patients with node-negative breast cancer for systemic adjuvant therapy. Expression of epidermal growth factor receptor (EGFr) has been shown previously to be inversely related to oestrogen receptor (ER) in patients with operable breast cancer and to be associated with a poorer prognosis. Analysis of EGFr and ER was performed on tumour samples from 231 patients with operable breast cancer followed for up to 6 years after surgery. The median duration of follow-up in patients still alive at the time of analysis was 45 months. Thirty-five percent of patients (82) had tumours with greater than 10 fmol mg$^{-1}$ $I^{125}$-EGF binding (EGFr +) and 47% (109) and cystolic ER concentrations $>5$ fmol mg$^{-1}$ (ER + ), with a marked inverse relationship between EGFr and ER ($P<0.00001$). In a univariate analysis EGFr was second only to axillary node status as a prognostic marker for all patients both in terms of relapse-free and overall survival ($P<0.001$, log rank). For patients with histologically negative axillary nodes EGFr was superior to ER in predicting relapse and survival ($P<0.01$ and $P<0.005$ respectively compared to $P<0.1$ and $P<0.1$, log rank). In a multivariate (Cox model) analysis only EGFr, out of EGFr, ER, size and grade, was predictive for either relapse-free or overall survival for patients with node-negative disease ($P=0.05$ and $P=0.026$ respectively). EGFr has been shown to be a marker of poor prognosis for patients with node-negative breast cancer. Since patients with EGFr + tumours are unlikely to respond to hormone therapy it may be possible to select them for trials of systemic adjuvant chemotherapy.

The histological status of the axillary lymph nodes, specifically the absolute number of nodes involved, remains the most potent prognostic marker for patients with operable breast cancer (Valagussa *et al.*, 1978; Fisher *et al.*, 1983). Tumour recurrence and death due to breast cancer, however, affects a significant proportion of patients with node-negative disease (Fisher *et al.*, 1989*c*). Recurrence rates of up to 43% and mortality at 10 years of 32% have been reported (Fisher *et al.*, 1989*b*), suggesting a need for effective adjuvant systemic therapy for selected patients in this supposedly good prognostic subgroup.

It is now recognised that there is a need for a marker (or markers) capable of discriminating patients with axillary node-negative disease at high risk of recurrence and death. Tumour oestrogen receptor (ER) content has been proposed but there is disagreement about its value (Cooke *et al.*, 1979; Fisher *et al.*, 1988). This has been supported by the long term results of large trials evaluating the use of adjuvant tamoxifen for patients with operable breast cancer in which a beneficial effect is shown also in patients with ER negative tumours (Nolvadex Adjuvant Trial Organisation [NATO], 1988). Although recent trials of systemic adjuvant chemotherapy in patients with axillary node-negative disease and ER negative tumours have shown small but significant benefits in terms of recurrence-free survival, there was no effect on overall survival (Fisher *et al.*, 1989*b*; Mansour *et al.*, 1989; Ludwig breast cancer study group, 1989) but this may reflect a relatively short follow-up. In one small trial there was a benefit in terms of overall survival (Bonadonna & Valagussa, 1987).

There have been several reports describing the presence of specific, high affinity receptors for epidermal growth factor (EGFr) on membranes prepared from primary human breast carcinoma (Sainsbury *et al.*, 1985; Perez *et al.*, 1984). These studies showed a marked inverse relationship between expression of EGFr and ER. Tumours which overexpressed EGFr were associated with a poor overall prognosis (Sainsbury *et al.*, 1987; Rios *et al.*, 1988) but short follow-up precluded close examination of patient subgroups in particular node-negative patients. Continued prospective patient follow-up and increased patient numbers now allows examination of the prognostic value of EGFr, particularly in node-negative patients.

Correspondence: S. Nicholson.
Received 31 May 1990; and in revised form 6 September 1990.

### Patients and methods

Two hundred and thirty-one consecutive patients with operatable breast cancer and without biochemical or radiological evidence of distant metastases were treated by simple mastectomy ($n=181$) or wide local excision and post-operative radiotherapy by external beam and iridium wire implants ($n=50$). Level I axillary nodes (behind pectoralis major) were sampled if palpable at surgery ($n=129$, 56%). An average of four nodes were sampled and for those with positive nodes an average of three contained metastatic tumour (range 1-8). In patients treated by mastectomy adjuvant radiotherapy was given to the ipsilateral axilla if the nodes were involved. No patient received adjuvant chemotherapy in this study. Forty patients towards the end of the study received adjuvant tamoxifen (20 mg daily) following the early results of the NATO study.

The resection specimens were taken immediately to the Department of Pathology on ice. Maximum tumour diameter was recorded and tumour blocks removed for histopathology. Tumour specimens adjacent to the block for histology were stored at $-20$°C in a sucrose/glycerol medium (Crawford *et al.*, 1984) prior to receptor analysis. EGFr was determined using an $I^{125}$-labelled EGF radioreceptor assay with a cut-off value of 10 fmol mg$^{-1}$ protein and ER by the dextran charcoal method with a 5 fmol mg$^{-1}$ cytosol protein cut-off as previously described (Nicholson *et al.*, 1988*b*). Histological examination of haematoxylin and eosin stained, formalin fixed, paraffin embedded sections was performed and ductal carcinomas graded according to the modified Bloom and Richardson method (Elston *et al.*, 1982).

Follow-up was conducted every 3 months for the first 18 months, 6 monthly until 3 years and annually thereafter until recurrence had been confirmed radiologically, cytologically or histologically. Follow-up thereafter was every 3 months or less until death. Patients with confirmed recurrent disease were treated according to standard protocols in an advanced medical oncoloy clinic (ALH). Patients with predominantly soft tissue or skeletal disease were treated by endocrine manipulation (tamoxifen if postmenopausal or oophorectomy ± tamoxifen if premenopausal). Patients with visceral disease and those who had failed first and second line endocrine therapy were treated with chemotherapy – usually single agent with either mitoxantrone or adriamycin. Patients with isolated soft tissue relapse (axilla or mastectomy flap) usually received radiotherapy in addition to endocrine manipulation.

### *Statistics*

Analysis of patient and tumour characteristics within subgroups was performed using Chi-square contingency tables. Survival data was analysed using the log rank method (Peto *et al.*, 1977) and the Cox proportional hazards regression model (Cox, 1972).

### Results

### *Relationship of EGFr to other prognostic indicators*

Of 231 patients, 213 (92%) had ductal carcinomas. Eighty-four patients (36%) were under 50 years of age and 147 (64%) were over 50. Eighty-two patients (35%) had tumours with levels of EGFr$>$10 fmol mg$^{-1}$ protein (EGFr positive) and 109 patients (47%) had tumours with a cytosolic ER content $>$5 fmol mg$^{-1}$ (ER positive). There was a marked inverse relationship between EGFr and ER ($P<0.00001$, Table I).

In the 213 patients with ductal carcinomas there was a significant association between increasing tumour grade and expression of EGFr ($P<0.025$, Table II). There was no relationship between EGFr and axillary node status (Table III) or tumour size (Table IV).

### *Relationship of EGFr to time to relapse and overall survival*

After a median follow-up of 45 months for patients still alive at the time of analysis, 125 patients (54%) have recurred and

**Table I** Relationship of tumour ER and EGFr expression in 231 patients with operable breast cancer

| | | *EGFr* − | *EGFr* + | | |
|---|---|---|---|---|---|
| *ER* | − | 55 | 67 | 122 | (53%) |
| | + | 94 | 15 | 109 | (47%) |
| | | 149 (65%) | 82 (35%) | 231 | |

$\chi^2 = 40.808$, dof = 1, $P<0.00001$.

**Table II** Relationship between tumour grade and EGFr expression in the 213 patients with ductal carcinomas

| | | *EGFr* − | *EGFr* + | | *% EGFr positive* |
|---|---|---|---|---|---|
| Bloom & Richardson | I | 23 | 4 | 27 | 15 |
| | II | 49 | 24 | 73 | 33 |
| | III | 63 | 50 | 113 | 44 |
| | | 135 | 78 | 213 | 37 |

$\chi^2 = 8.805$, dof = 2, $P<0.025$.

**Table III** Relationship between EGFr and axillary node status according to clinical or histopathological assessment

| A *Clinical* | | *EGFr* − | *EGFr* + | |
|---|---|---|---|---|
| Node | − | 105 | 47 | 152 |
| | + | 44 | 35 | 79 |
| | | 149 | 82 | 231 |
| | | $\chi^2 = 3.5$, $P$ 0.06 | | |
| **B** *Histological* | | *EGFr* − | *EGFr* + | |
| Node | − | 30 | 20 | 50 |
| | + | 44 | 35 | 79 |
| | | 74 | 55 | 129 |
| | | NS | | |

(A) Histopathological; (B) assessment.

**Table IV** Relationship between EGFr and tumour size

| | | *EGFr* − | *EGFr* + | | | *% EGFr positive* |
|---|---|---|---|---|---|---|
| Size | $T_1$ | 36 | 17 | 53 | | 32 |
| | $T_2$ | 96 | 54 | 150 | NS | 36 |
| | $T_3$ | 17 | 11 | 28 | | 39 |
| | | 149 | 82 | 231 | | 35 |

**Figure 1** Survival of patients with operable breast cancer stratified by tumour EGFr expression; **a**, relapse-free, **b**, overall survival.

there have been 80 breast cancer related deaths (35%). Fifty-five patients out of 82 with EGFr positive tumours have recurred compared with 70/149 with EGFr negative tumours ($P<0.001$, log rank, Figure 1a). Forty-one patients out of 82 with EGFr positive tumours have died compared with 39/149 with EGFr negative tumours ($P<0.001$, log rank Figure 1b). In a univariate analysis of prognostic factors in all 231 patients EGFr was second only to axillary node status (clinical assessment) considering all patients (Table Va) or those with histologically confirmed axillary node status (Table Vb).

The effect of EGFr overexpression in specific patient subgroups is shown in Table Vc. In the 50 patients with histologically examined and negative axillary nodes EGFr expression was associated with a significant reduction in recurrence-free and overal survival ($P<0.01$ and $<0.005$ respectively – Figure 2a and b). Similarly in patients with lower grade tumours (Grades I and II) there was a reduction in recurrence-free and overall survival for those with EGFr positive tumours (Figures 3a and b).

For patients with ER negative tumours co-expression of EGFr reduced survival (Figure 4a). There was a similar trend for patients whose tumours contained both EGFr and ER (Figure 4b) but this did not reach statistical significance. None of the factors analysed were of significance for patients with positive nodes, although median relapse-free and overall survival were approximately 10 months greater for patients with node positive, EGFr negative tumours compared to node positive, EGFr positive tumours by a univariate (log rank) analysis.

### *Multivariate analysis of survival data*

A multivariate analysis was performed using a stepwise regression (Cox) model. Five factors were assessed in this analysis: axillary node status, tumour grade, tumour size (analysed as a continuous variable only), EGFr and ER. This allowed, therefore, analysis of only the 213 patients (92%) with ductal carcinomas. The results are summarised in Table VI. In the first analysis all the factors were assessed in all 213 patients (Table VI*a*). Clinical axillary node status was either positive (sampled and histologically positive) or not positive (sampled and histologically negative or unsampled). EGFr was the only significant variable for recurrence-free survival but just failed to reach significance for overall survival ($P=0.059$). Tumour size showed a trend only in terms of recurrence-free survival. Neither ER nor tumour grade were

**Table V** Univariate analysis of survival data (log rank)

| | | | |
|---|---|---|---|
| A | *Factor* | *Disease-free* | *Overall survival* |
| | Axillary nodes | $P<0.005$ | $P<0.001$ |
| | EGFr | $P<0.001$ | $P<0.001$ |
| | ER | $P<0.01$ | $P<0.01$ |
| | Tumour grade | NS | $P<0.025$ |
| | Size | NS | $P<0.1$ |
| B | | *Disease-free* | *Overall survival* |
| | Axillary nodes | $P<0.005$ | $P<0.001$ |
| | EGFr | $P<0.01$ | $P<0.01$ |
| | ER | NS | $P<0.1$ |
| | Tumour grade | NS | NS |
| | Size | NS | NS |
| C | | *Disease-free* | *Overall survival* |
| | Axillary node negative | $P<0.01$ | $P<0.005$ |
| | Bloom & Richardson | | |
| | Grade I | $P<0.01$ | $P<0.005$ |
| | Grade II | $P<0.005$ | $P<0.001$ |
| | ER + | $P<0.05$ | $P<0.1$ |
| | ER – | $P<0.1$ | $P<0.05$ |

A: Effect of prognostic factors on survival of 231 patients; B: Axillary node status known; C: Effect of overexpression of EGFr on survival of patient subgroups.

**Figure 2** Survival of patients with histologically negative axillary nodes stratified by tumour EGFr expression; **a**, relapse-free, **b**, overall survival.

**Figure 3** Survival of patients with **a**, low and **b**, intermediate grade tumours stratified by tumour EGFr expression.

**Figure 4** **a**, Survival of patients with ER − tumours stratified by EGFr. **b**, Survival of patients with ER + tumours stratified by EGFr.

**Table VI** Multivariate analysis (Cox model) of data for recurrence-free and overall survival of 213/231 patients with ductal carcinomas

| | Covariate | Recurrence | Death |
|---|---|---|---|
| A | *213 patients with ductal carcinoma* | | |
| | Lymph node | *P* 0.124 | *P* 0.0005 |
| | EGFr | *P* 0.031 | *P* 0.059 |
| | ER | NS | NS |
| | Bloom & Richardson | NS | *P* 0.142 |
| | Size | *P* 0.055 | NS |
| B | *121 patients with ductal carcinomas and histological node status* | | |
| | Lymph node | *P* 0.027 | *P* 0.002 |
| | EGFr | *P* 0.053 | *P* 0.072 |
| | ER | NS | NS |
| | Bloom & Richardson | NS | NS |
| | Size | *P* 0.116 | NS |
| C | *49 patients with ductal carcinomas and histologically negative axillary nodes* | | |
| | EGFr | *P* 0.05 | *P* 0.026 |
| | ER | NS | NS |
| | Bloom & Richardson | NS | NS |
| | Size | *P* 0.01 | NS |

A: All patients ($n = 213$); B: Patients with histologically confirmed node status ($n = 121$); C: Patients with histologically negative nodes ($n = 49$).

significant variables. In terms of overall survival only lymph node status was significant.

In the second analysis (Table VIb), 121 patients with histologically confirmed nodal status were assessed and the results were similar to the first analysis with axillary nodal status emerging as the most powerful prognostic marker for both recurrence-free and overall survival ($P = 0.027$ and $P = 0.002$ respectively). EGFr just failed to reach significance in this analysis ($P = 0.053$ and $P = 0.072$ respectively). Neither ER, tumour grade nor size were significant factors.

In the third analysis (Table VIc) only patients with axillary node-negative (histologically confirmed), ductal carcinomas ($n = 49$) were assessed. Both EGFr and tumour size were significant factors for recurrence-free survival but only EGFr was significant for overall survival.

### Discussion

This study has emphasised the value of EGFr in selecting a poor prognosis group within a population of patients with operable breast cancer. The findings in the node negative group are potentially of greater importance. The study, however, was conducted between 1983 and 1987 before the worldwide results of adjuvant therapy were collated. During this period it was the policy of many British surgeons to perform only selective node sampling whereas now most authorities would consider node sampling essential. In this paper we have emphasised the analyses where node status was histologically confirmed in order not to overstate the extent of the findings.

There can be little doubt that patients with involved axillary lymph nodes require adjuvant therapy but debate continues as to the optimum. Systemic adjuvant chemotherapy almost certainly confers a survival advantage in premenopausal patients (Fisher *et al.*, 1986; Bonadonna *et al.*, 1985) but the mechanism of action has been questioned (Padmanabhan *et al.*, 1986). In the postmenopausal node-positive patient, tamoxifen therapy for 2 or more years significantly improves survival (Nolvadex Adjuvant Trial Organisation, 1988; Breast Cancer Trials Committee; Scottish Cancer Trials Office (MRC), 1987) regardless of ER status. There is increasing evidence that some node-negative patients should also receive adjuvant therapy but the criteria for selection are not clear. A recent trial of adjuvant tamoxifen in node-negative patients with ER positive tumours (Fisher *et al.*, 1989*a*) showed an advantage in the treated group in terms of disease-free but not overall survival.

These studies suggest that, although ER may be a useful prognostic marker overall (Cooke *et al.*, 1979) and in predicting response to endocrine therapy at relapse (Leake *et al.*, 1981), it may not be so useful in assigning different patient subgroups to the currently available adjuvant therapies.

Thymidine labelling has been used as a prognostic marker in node-negative disease (Silvestrini *et al.*, 1985) but the technique is complex and time consuming and, therefore, probably not applicable to routine clinical practice. A recent study (Clark *et al.*, 1989) showed that flow cytometry may offer a practical alternative. In this study disease-free survival for patients with node-negative breast cancer was significantly worse if their tumours were either aneuploid or diploid with a high S-phase fraction.

Expression of the c-*erb*B-2 oncoprotein, which can be measured using immunohistochemical staining of formalin fixed, paraffin embedded sections, has recently been evaluated as a prognostic marker in breast cancer with promising results (Wright *et al.*, 1989), but its prognostic power may be greatest in node-positive patients (Slamon *et al.*, 1987) from whom few clinicians would now withhold adjuvant therapy.

Overexpression of EGFr has been shown previously to be a marker of early relapse and death in patients with operable breast cancer (Sainsbury *et al.*, 1987; Rios *et al.*, 1988). Overexpression of EGFr in breast tumours of elderly patients treated with primary endocrine therapy was associated with rapid disease progression and poor response rates (Nicholson *et al.*, 1988*a*) as in patients with recurrent breast cancer after surgical treatment (Nicholson *et al.*, 1989). For these reasons we felt it was appropriate to include the small number of patients accrued at the end of the study who received adjuvant tamoxifen, since the survival of at least those with EGFr positive tumours was unlikely to be influenced.

This study has demonstrated that overexpression of EGFr in tumours of patients with operable breast cancer is associated with a poor overall prognosis and that EGFr status is second only to axillary lymph node status in its prognostic power. In subgroups which would otherwise have been considered to have a good prognosis (axillary node-negative and low tumour grade) overexpression of EGFr led to a significant reduction in both relapse-free and overall survival. Similarly, patients whose tumours coexpressed EGFr and ER had survival patterns similar to those expressing EGFr alone, suggesting that overexpression of EGFr conferred a growth potential on a tumour which obviated its requirement for oestrogen. Although the node negative (histologically confirmed) groups was relatively small the magnitude of the differences in this group between those with EGFr negative and positive tumours was great. In a multivariate analysis when clinical node status was assessed the effect of EGFr expression in the node negative group was enhanced ($P = 0.01$ for relapse-free and overall survival) suggesting that these differences are indeed of practical importance.

These results, together with those which have shown a failure of response to endocrine therapy associated with overexpression, suggest that EGFr may be a clinically useful prognostic marker in patients with axillary node-negative breast cancer capable of identifying a poor prognosis subgroup which may benefit from systemic adjuvant chemotherapy.

### References

BONADONNA, G., ROSSI, A. & VALAGUSSA, P. (1985). Adjuvant CMF chemotherapy in operable breast cancer: Ten years later. *Lancet*, **i**, 976.

BONADONNA, G. & VALAGUSSA, P. (1987). Current status of adjuvant chemotherapy for breast cancer. *Seminars in Oncol.*, **14**, 8.

BREAST CANCER TRIALS COMMITTEE; SCOTTISH CANCER TRIALS OFFICE (MRC), EDINBURGH (1987). Adjuvant tamoxifen in the management of operable breast disease: The Scottish trial. *Lancet*, **ii**, 171.

CLARK, G.M., DRESSLER, L.G., OWENS, M.A., POUNDS, G., OLDAKER, T. & MCGUIRE, W.L. (1989). Prediction of relapse or survival in patients with node-negative breast cancer by DNA flow cytometry. *N. Engl. J. Med.*, **320**, 627.

COOKE, T., SHIELDS, R., GEORGE, D., MAYNARD, P. & GRIFFITHS, K. (1979). Oestrogen receptors and prognosis in early breast cancer. *Lancet*, **i**, 995.

COX, D.R. (1972). Regression models and life tables. *J. R. Stat. Soc. (B)*, **34**, 187.

CRAWFORD, D., COWANS, S., HYDER, S., MCMENAMIN, M., SMITH, D. & LEAKE, R. (1984). Tumour biopsies prior to oestrogen receptor measurement. *Cancer Res.*, **44**, 2348.

ELSTON, C.W., GRESHAM, G.A., RAO, G.S. & 4 others (1982). The Cancer Research Campaign (King's/Cambridge) trial for early breast cancer; clinicopathological aspects. *Br. J. Cancer*, **45**, 655.

FISHER, B., BAUER, M., WICKERHAM, L., REDMOND, C.K. & FISHER, E.R. (1983). Relation of number of positive axillary nodes to the prognosis of patients with primary breast cancer, an NSAPB update. *Cancer*, **52**, 1551.

FISHER, B., COSTRANTINO, J., REDMOND, C. & 17 others (1989*a*). A randomized clinical trial evaluating tamoxifen in the treatment of patients with node-negative breast cancer who have estrogen-receptor-positive tumours. *N. Engl. J. Med.*, **320**, 479.

FISHER, B., REDMOND, C., DIMITROV, N. V. & 10 others (1989*b*). A randomized clinical trial evaluating sequential methotrexate and fluorouracil in the treatment of patients with node-negative breast cancer who have estrogen-receptor-negative tumour. *N. Engl. J. Med.*, **320**, 473.

FISHER, B., REDMOND, C., FISHER, E. & 12 others (1988). Relative worth of estrogen or progesterone receptor and pathologic characteristics of differentiation as indicators of prognosis in node-negative breast cancer patients: findings from National Surgical Adjuvant Breast and Bowel Project B-06. *J. Clin. Oncol.*, **6**, 1076.

FISHER, B., REDMOND, C., FISHER, E.R. & WOLMARK, N. (1986). Systemic adjuvant therapy in treatment of primary operable breast cancer: National Surgical Adjuvant Breast and Bowel Project. *Natl Cancer Inst.* Monogr., **1**, 35.

FISHER, B., REDMOND, C.K., POISSON, R. & 12 others (1989*c*). Eight-year results of a randomized clinical trial comparing total mastectomy and lumpectomy with or without irradiation in the treatment of breast cancer. *N. Engl. J. Med.*, **320**, 822.

LEAKE, R.E., LAING, L., CALMAN, K.C., MACBETH, F.R., CRAWFORD, D. & SMITH, D.C. (1981). Oestrogen receptor status and endocrine therapy of breast cancers: response rates and status stability. *Br. J. Cancer*, **43**, 59.

LUDWIG BREAST CANCER STUDY GROUP (1989). Prolonged disease-free survival after one course of perioperative adjuvant chemotherapy for node-negative breast cancer. *N. Engl. J. Med.*, **320**, 491.

MANSOUR, E.G., GRAY, R., SHATILA, A.H. & 5 others (1989). Efficacy of adjuvant chemotherapy in high-risk node-negative breast cancer. *N. Engl. J. Med.*, **320**, 485.

NICHOLSON, S., HALCROW, P., SAINSBURY, J.R.C. & 4 others (1988*a*). Epidermal growth factor receptor (EGFr) status associated with failure of primary endocrine therapy in elderly postmenopausal patients with breast cancer. *Br. J. Cancer*, **58**, 810.

NICHOLSON, S., SAINSBURY, J.R.C., HALCROW, P., CHAMBERS, P., FARNDON, J.R. & HARRIS, A.L. (1989). Expression of epidermal growth factor receptors associated with lack of response to endocrine therapy in recurrent breast cancer. *Lancet*, **i**, 182.

NICHOLSON, S., SAINSBURY, J.R.C., NEEDHAM, G.K., CHAMBERS, P., FARNDON, J.R. & HARRIS, A.L. (1988*b*). Quantitative assays of epidermal growth factor receptor in human breast: Cut off points of clinical relevance. *Int. J. Cancer*, **42**, 36.

NOLVADEX ADJUVANT TRIAL ORGANISATION (1988). Controlled trial of tamoxifen as single adjuvant agent in the management of early breast cancer: analysis at eight years by 'Nolvadex' Adjuvant Trial Organisation. *Br. J. Cancer*, **57**, 608.

PADMANABHAN, N., HOWELL, A. & RUBENS, R. (1986). Mechanisms of action of adjuvant chemotherapy in early breast cancer. *Lancet*, **ii**, 411.

PEREZ, R., PASCUAL, M., MACIAS, A. & LAGE, A. (1984). Epidermal growth factor receptors in human breast cancer. *Breast Cancer Res. Treat.*, **4**, 189.

PETO, R., PYKE, M.C. & ARMITAGE, N.E. (1977). Design and analysis of randomized clinical trials requiring prolonged observation of each patient. II Analysis and examples. *Br. J. Cancer*, **35**, 1.

RIOS, M.A., MACIAS, A., PEREZ, R., LAGE, A. & SKOOG, L. (1988). Receptors for epidermal growth factor and estrogen as predictors of relapse in patients with mammary carcinoma. *Anticancer Res.*, **8**, 173.

SAINSBURY, J.R.C., FARNDON, J.R., NEEDHAM, G.K., MALCOLM, A.J. & HARRIS, A.L. (1987). Epidermal growth factor receptor status as predictor of early recurrence of and death from breast cancer. *Lancet*, **i**, 1398.

SAINSBURY, J.R.C., SHERBERT, G.V., FARNDON, J.R. & HARRIS, A.L. (1985). Epidermal growth factor receptors and oestrogen receptors in human breast cancer. *Lancet*, **i**, 364.

SILVESTRINI, R., DIADONE, M.G. & GASPAIRINI, G. (1985). Cell kinetics as a persistent prognostic marker in node-negative breast cancer. *Cancer*, **56**, 1982.

SLAMON, D.J., CLARK, G.M., WONG, S.G., LEVIN, W.J., ULLRICH, A. & MCGUIRE, W.L. (1987). Human breast cancer: correlation of relapse and survival with amplification of the HER-2/*neu* oncogene. *Science*, **235**, 177.

VALAGUSSA, P., BONADONNA, G. & VERONESI, U. (1978). Patterns of relapse and survival following radical mastectomy: Analysis of 716 consecutive patients. *Cancer*, **41**, 1170.

WRIGHT, C., ANGUS, B., NICHOLSON, S. & 6 others (1989). Expression of c-*erb*B-2 oncoprotein: a prognostic marker in human breast cancer. *Cancer Res.*, **49**, 2087.